# Functional Outcomes of Intramedullary Interlocking Nailing for Humeral Shaft Fractures: A Prospective Study

**DOI:** 10.7759/cureus.82550

**Published:** 2025-04-18

**Authors:** Challa Veera Naveen Kumar Reddy, Sandeep Naik, Ravi Kumar Biradar

**Affiliations:** 1 Department of Orthopedics, Shri B M Patil Medical College, Hospital and Research Centre, BLDE (Deemed to Be University), Vijayapura, IND

**Keywords:** dash score, functional outcomes, humerus nailing, humerus shaft fractures, intramedullary interlocking nail

## Abstract

Background

Humeral shaft fractures were frequent orthopedic injuries, typically caused by trauma, and the optimal treatment approach remained a subject of discussion. Intramedullary interlocking (IMIL) nailing was a minimally invasive surgical method that offered superior biomechanical stability, especially for diaphyseal fractures, while promoting early mobilization. This prospective study assessed the functional outcomes of IMIL nailing in managing humeral shaft fractures.

Objectives

This study aims to evaluate the functional outcomes of IMIL nails in treating closed humeral shaft fractures and to analyze common complications associated with humeral nailing, including non-union rates, infection, shoulder impingement, and the necessity for secondary procedures.

Materials and methods

We included 40 cases of humeral shaft fractures managed with IMIL nails. Patients were followed for a minimum of six months. The inclusion criteria included individuals aged 18 to 70 with closed or segmental fractures. Functional outcomes were assessed using the Disabilities of the Arm, Shoulder, and Hand (DASH) score, while radiological union, intraoperative findings, and complications were recorded.

Results

Fracture union was achieved in 38 (95%) patients, with an average healing time of 10 to 16 weeks. At the final follow-up, the mean DASH score was 16.51, reflecting satisfactory arm, elbow, and shoulder function. Among the 40 cases, six (15%) demonstrated excellent functional outcomes, 26 (65%) had good results, six (15%) showed fair outcomes, and two (5%) experienced poor results, requiring secondary procedures to achieve union.

Conclusion

IMIL nailing was a reliable and effective method for managing humeral shaft fractures, providing excellent functional recovery, a high fracture healing rate, and manageable complications. Its advantages included early mobilization and minimal surgical morbidity, making it a viable treatment choice. The procedure led to significant functional improvement, as demonstrated by enhanced DASH scores at six months, underscoring its effectiveness as a minimally invasive approach with favorable long-term outcomes.

## Introduction

Humeral shaft fractures were among the most frequently encountered upper limb fractures, comprising 20% of all humeral fractures and 1-5% of all fractures in the body. Additionally, 2% to 5% of diaphyseal humerus fractures were open fractures. The annual incidence rose with age, ranging from 13 to 20 cases per 100,000 individuals [1].

The age distribution of humeral shaft fractures followed a bimodal pattern. The first peak occurred in males aged 21 to 30 years, typically resulting from high-energy trauma, often leading to comminuted fractures and associated soft tissue injuries. The second peak was seen in women aged 60 to 80, usually following low-energy trauma.

Traditionally, closed humeral shaft fractures were regarded as relatively benign, with a high primary healing rate when managed conservatively using a functional brace or a hanging arm cast. Malunion typically occurs due to inadequate reduction provided by the plaster cast [2].

Surgical treatment of humeral shaft fractures was typically performed using open reduction with plates and screws or intramedullary nails. However, plate and screw fixation was associated with complications such as extensive soft tissue stripping, radial nerve injury, significant blood loss, and an increased risk of infection [3].

Intramedullary interlocking (IMIL) nailing offered an alternative that avoided the complications associated with plate fixation and was particularly beneficial in cases of osteoporosis, where plate fixation carried a higher risk of implant failure. However, intramedullary nailing was associated with a greater incidence of shoulder pain and a higher non-union rate [4]. Due to these potential complications, IMIL nailing was typically reserved for treating segmental or pathological fractures or fractures with significant comminution.

In recent decades, advancements in humeral nail design, surgical techniques, and IMIL nailing have sparked interest in considering it as a primary treatment option for humeral shaft fractures.

This prospective study evaluated the functional outcomes of intramedullary nailing in closed humeral shaft fractures and examined its associated complications, aiming to support both recent and existing literature.

## Materials and methods

We conducted a prospective study on patients diagnosed with humeral shaft fractures who were admitted to the Orthopedics Department at Shri B M Patil Medical College, Hospital and Research Centre, BLDE (Deemed to Be University), Vijayapura, India, between March 2023 and March 2025. Approval was obtained from the Institutional Ethics Committee (IEC) at BLDE (Deemed to be University), under approval number BLDE(DU)/IEC/978/2022-23. The study involved 40 patients, including 24 (60%) males and 16 (40%) females. Of these, 25 (62.5%) patients had sustained a right-sided injury, while 15 (37.5%) had a left-sided injury. A minimum follow-up period of six months was completed for all patients.

Inclusion criteria

Patients aged 18 to 70 years with closed fractures and segmental fractures were included.

Exclusion criteria

Patients under 18 years of age or over 70 years, those with fractures at the proximal or distal ends of the humerus, pathological fractures, compound fractures, polytrauma, evidence of neurological or vascular disorders, and those with associated radial nerve injuries were excluded.

Eligible patients were enrolled after obtaining written informed consent. A thorough medical history was obtained, followed by a detailed clinical examination. Any radial nerve injury was noted and recorded if present. All patients with humeral shaft fractures were treated using the anterolateral approach with intramedullary nailing.

Surgical procedure

Preoperative Planning

Upon admission, patients underwent a thorough assessment of their medical history, physical examination, systemic evaluation, and general health status. X-ray imaging in both anteroposterior and lateral views was performed to assess the injured arm and confirm the diagnosis. Analgesics were provided, and the affected arm was immobilized using a Plaster of Paris (POP) U-slab. Patients were scheduled for surgery as soon as possible after completing preoperative hematological and other necessary tests.

Preoperative measurements of the humeral length and the width of the narrowest part of the humeral canal, with X-ray magnification adjustments, were used to select the appropriate nail length and diameter. Additional X-ray scans were obtained if the fracture lines extended toward the elbow or shoulder joints.

Positioning

**Figure 1:**
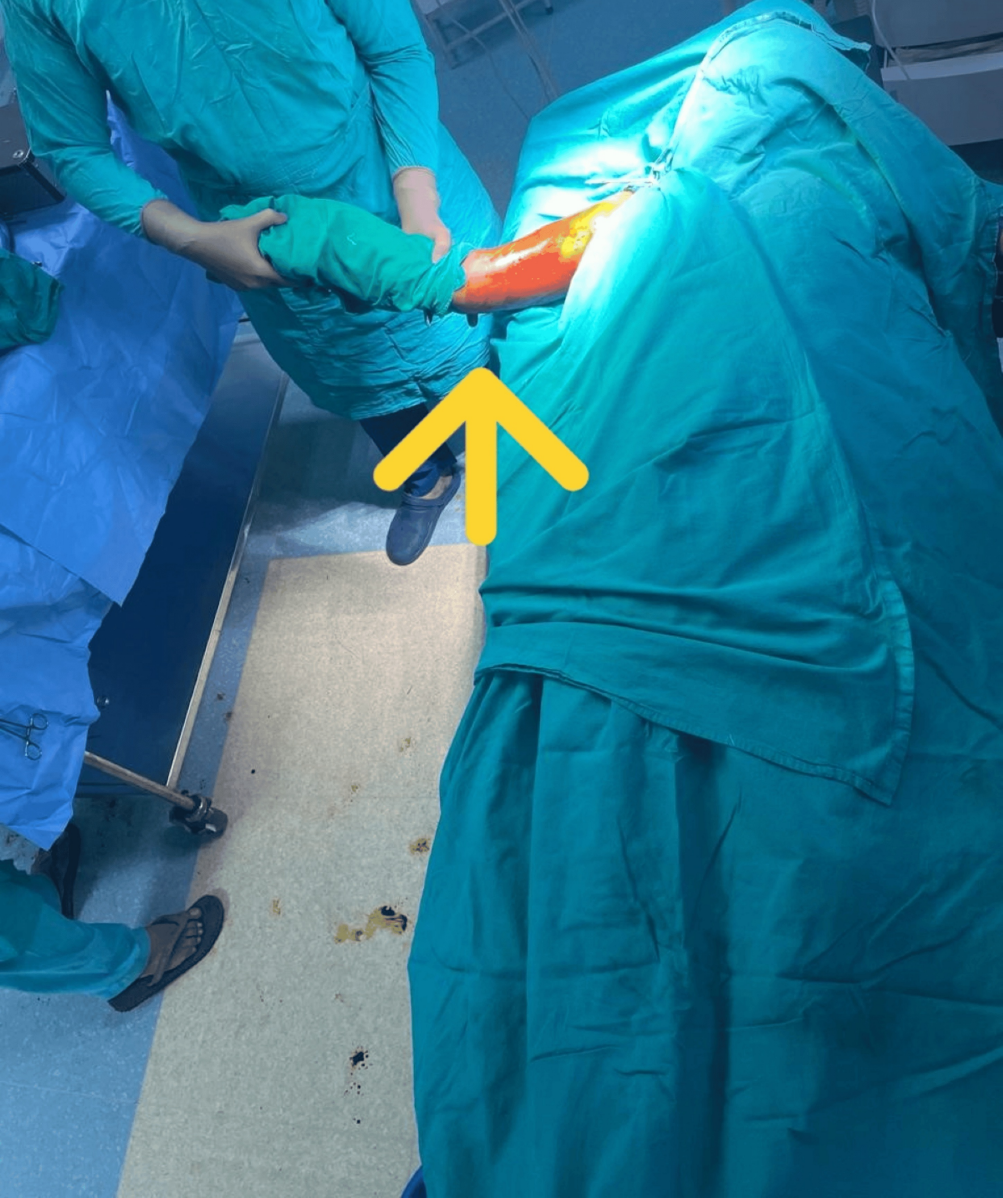
Patient positioning The arrow indicates that the sterile field encompassed the elbow, humerus, and shoulder.

The patients were placed in the supine position with padded support beneath the shoulder. The injured arm was allowed to hang off the side of the table while the torso was positioned on the operating table, as demonstrated in Figure 1. The sterile field encompassed the elbow, humerus, and shoulder, with the image intensifier positioned on the opposite side of the surgical field. We effectively stabilized humeral shaft fractures using durable Russell-Taylor interlocking nails, which featured a tapered tip for easier insertion and allowed smooth passage through the medullary canal.

Anterolateral Approach

**Figure 2:**
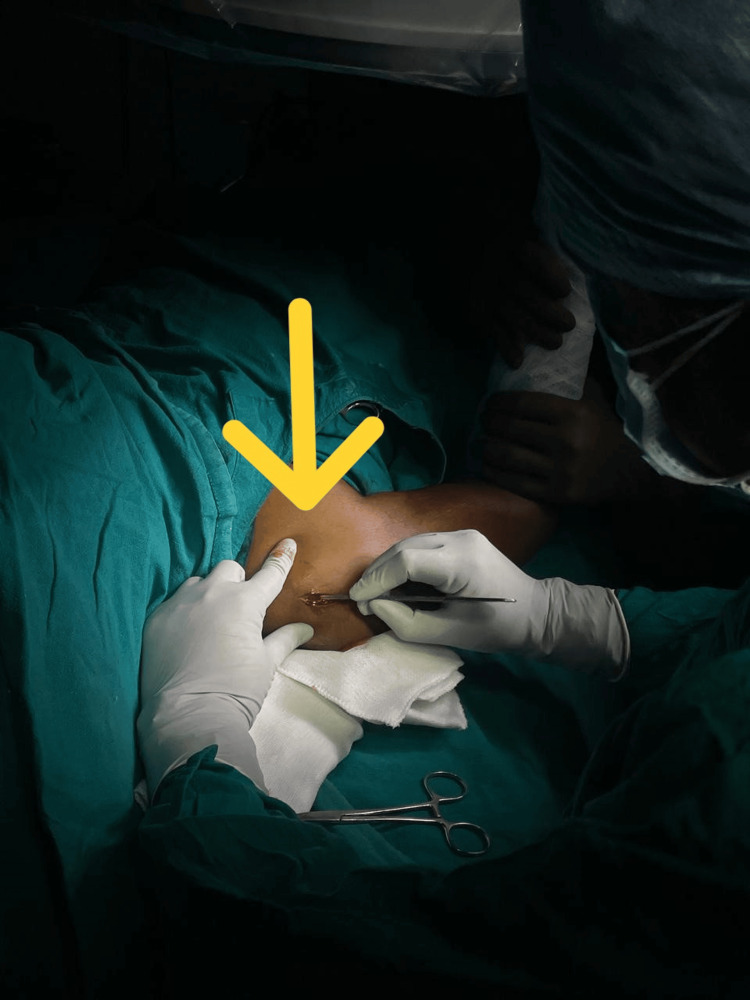
Skin incision The arrow markes the intraoperative view of the skin incision.

A 2- to 3-cm incision was made obliquely along the anterolateral border after locating the acromion. The deltoid muscle was then split along its fibers to expose the subacromial bursa and rotator cuff, as shown in Figure 2 [1].

**Figure 3:**
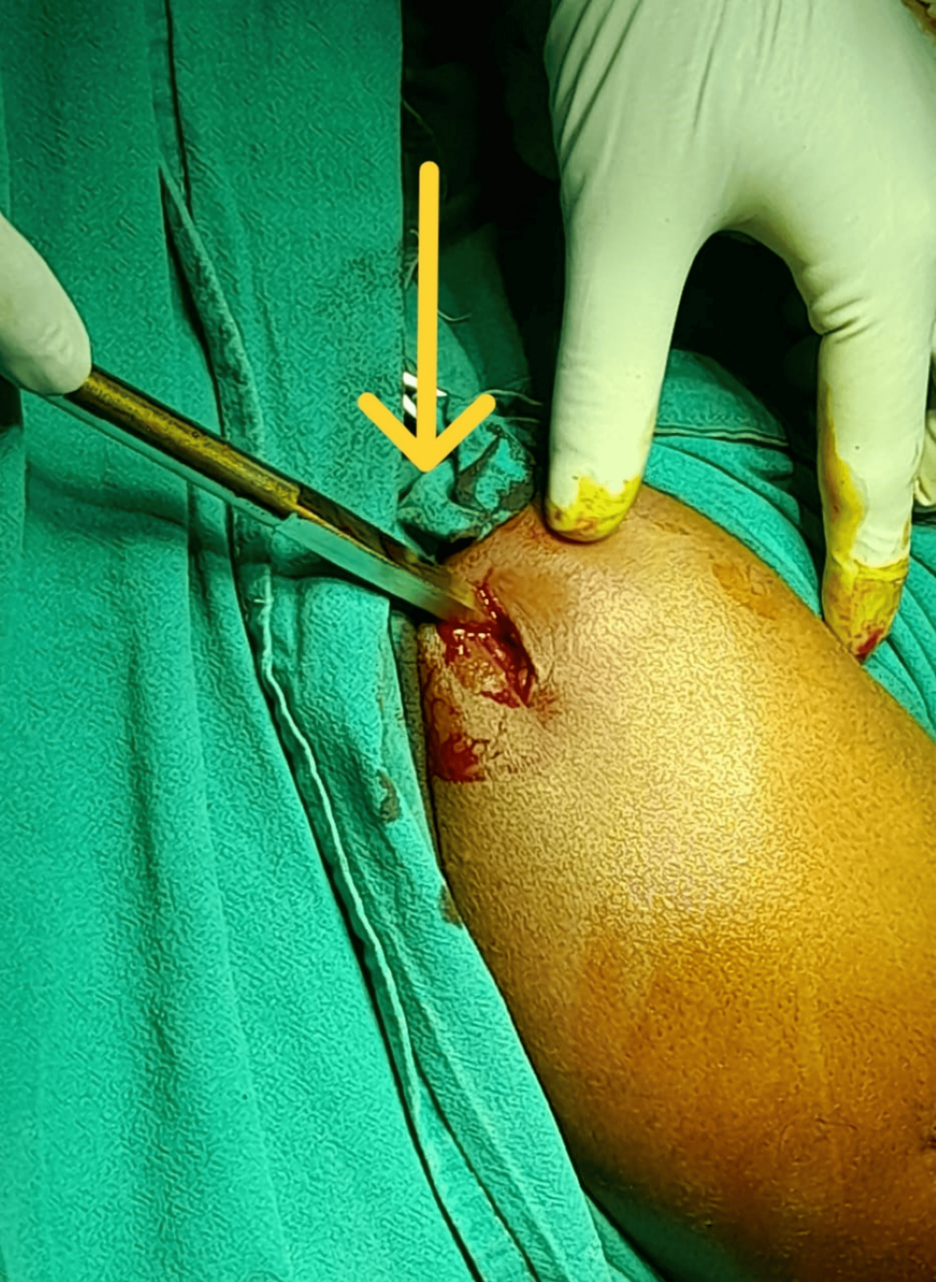
Awl entry The arrow markes the intraoperative image showing the use of a hand awl to create the entry portal for standard antegrade nailing.

A hand awl was used to create the entry portal for standard antegrade nailing. The awl was required to penetrate 4-5 cm into the humeral head to make space for the guidewire. The assistant maintained 90 degrees of elbow flexion, supination of the forearm, and traction to ensure proper arm alignment, as illustrated in Figure 3.

**Figure 4:**
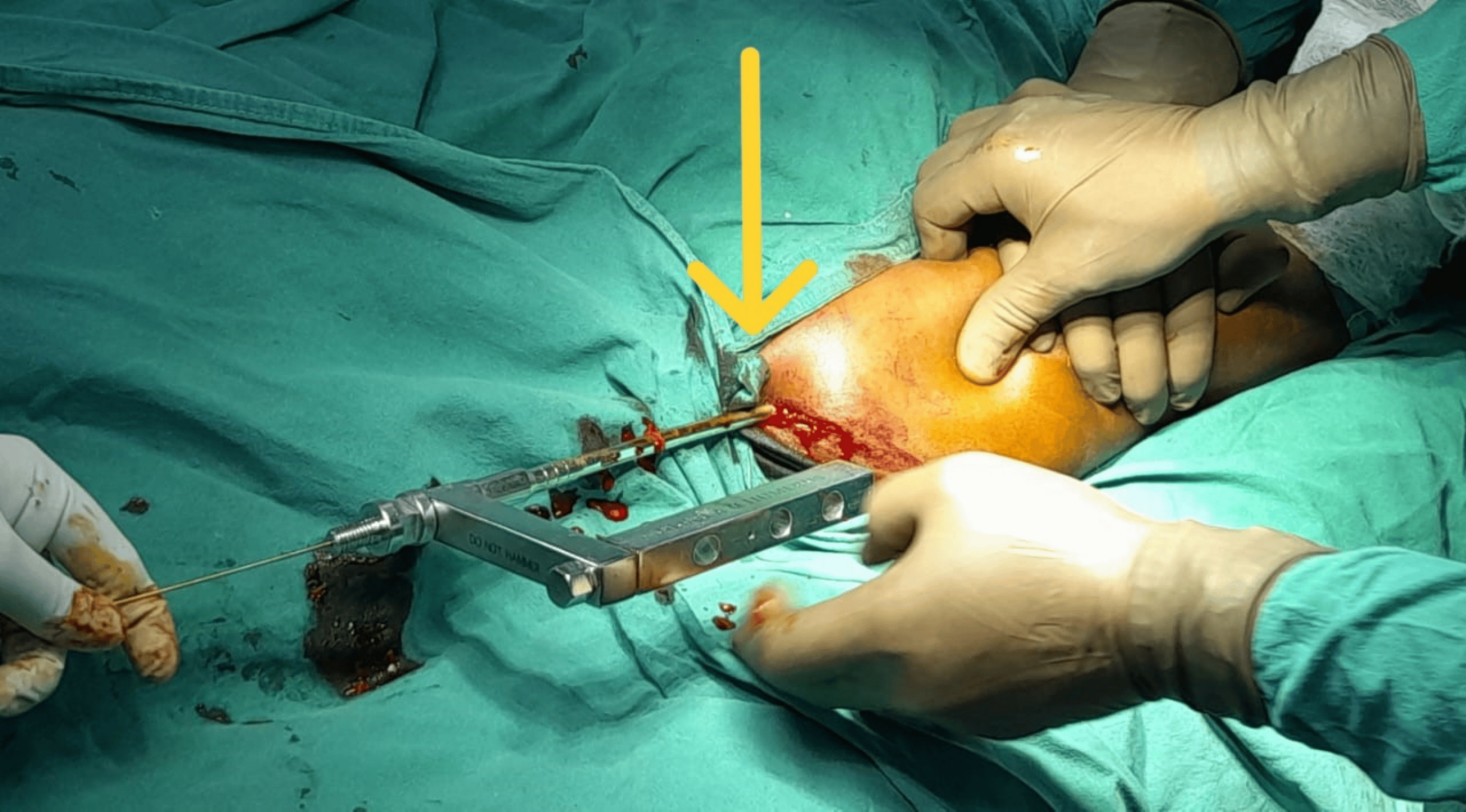
Nail insertion along the guide wire The arrow indicates the nail insertion following the passage of the guidewire.

Achieving a lateral view of the fracture site was challenging, so the guidewire’s passage into the distal fragment was confirmed by rotating the arm 40 to 50 degrees or by allowing slight angulation at the fracture site, as demonstrated in Figure 4.

**Figure 5:**
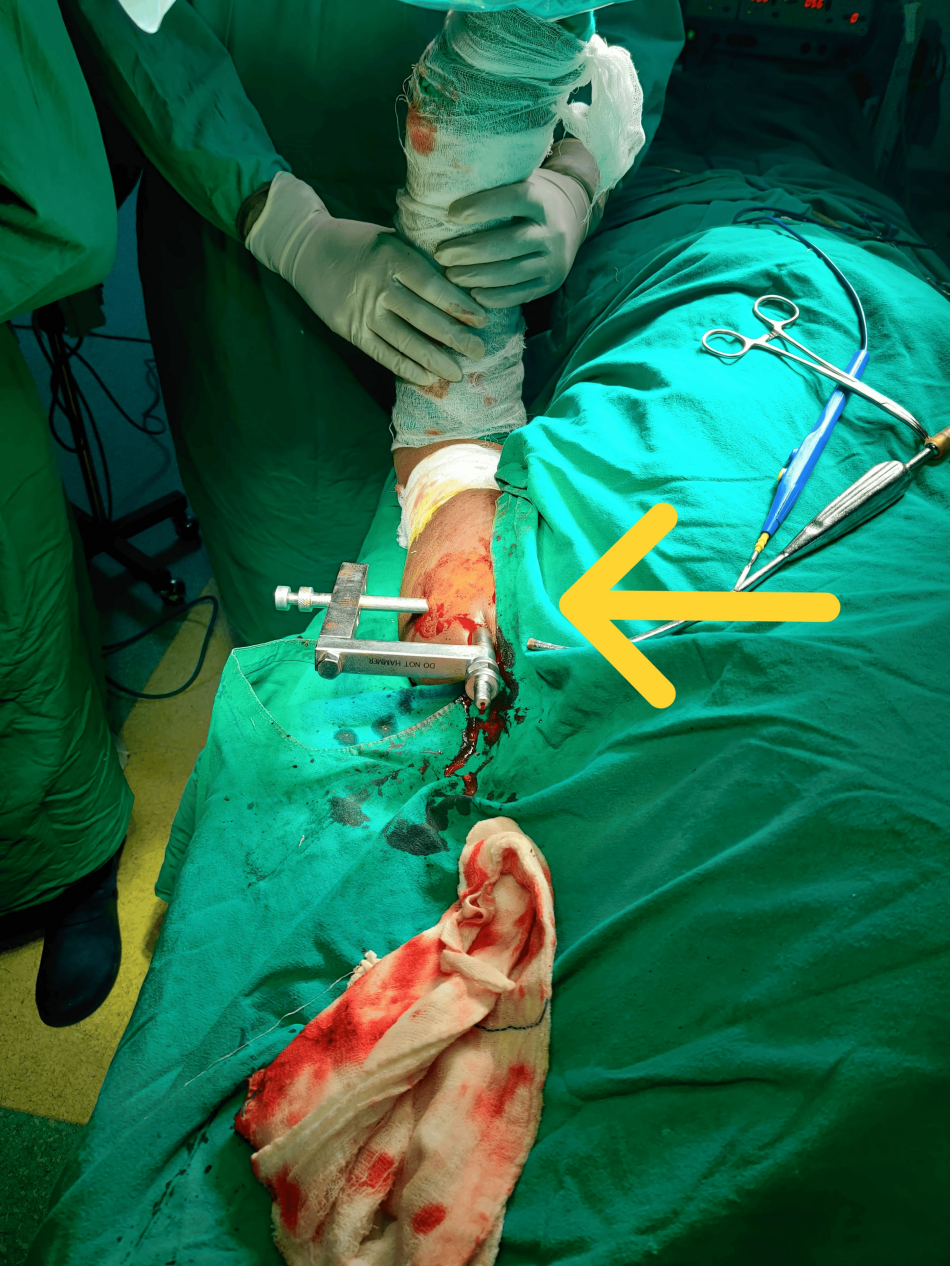
Proximal locking The arrow indicates the intraoperative image of proximal locking performed with the help of a sleeve.

After the nail was inserted into its final position, it was locked to provide adequate proximal and distal stability, as shown in Figure 5.

**Figure 6:**
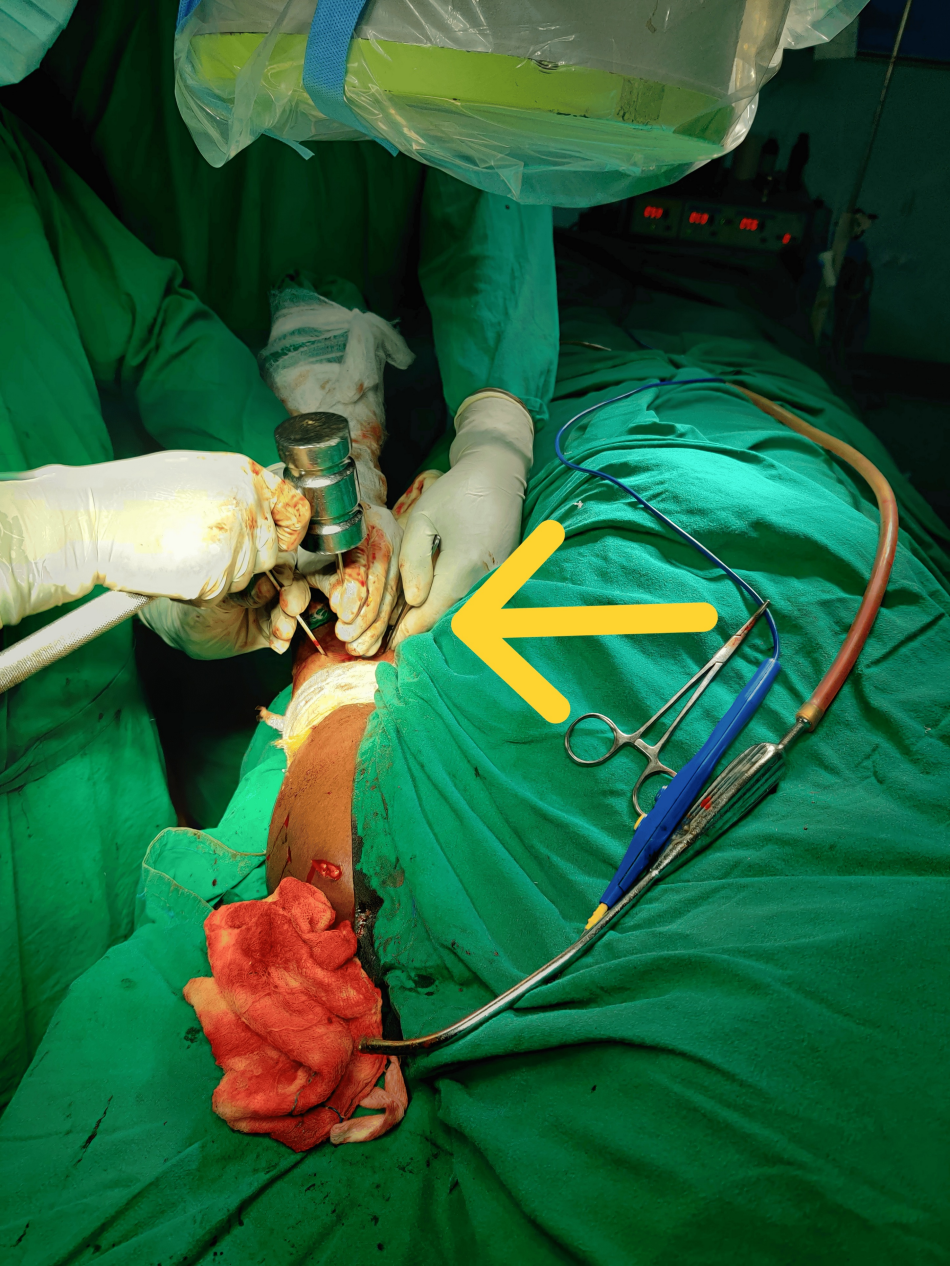
Distal locking The arrow indicates the intraoperative image of distal locking with the freehand technique.

The distal incision was made with care taken to avoid neurovascular structures, and distal locking was performed using the "freehand" technique, as shown in Figure 6.

**Figure 7:**
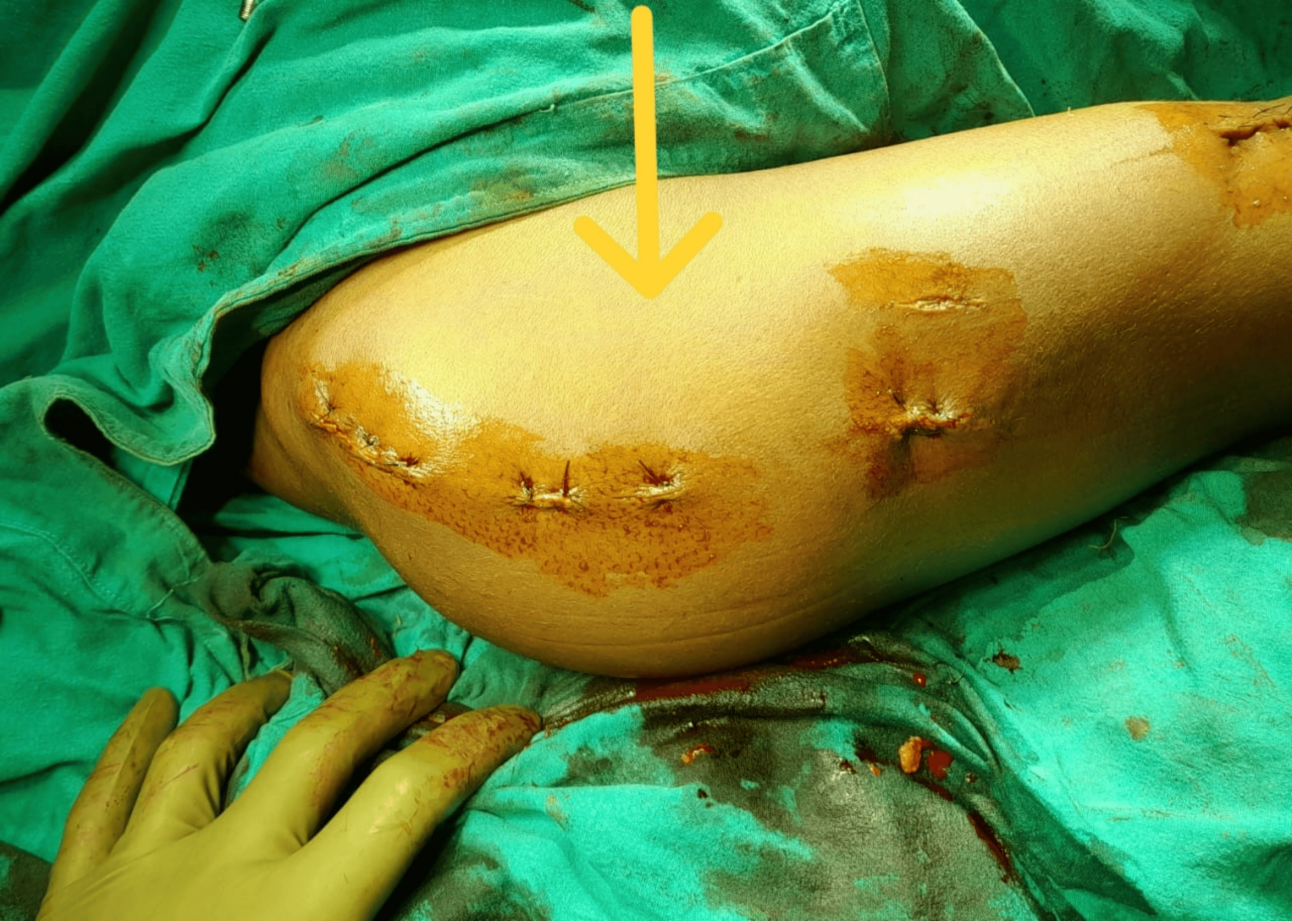
Wound closure The arrow indicates the intraoperative image showing the closure of the surgical site.

The rotator cuff and deltoid muscle were sutured with absorbable stitches, and the superficial layers were then closed, as illustrated in Figure 7.

Postoperative Management

After surgery, all patients were advised to support the affected arm with a collar and cuff to ensure comfort and maintain proper alignment during the early recovery period. In cases involving comminuted fractures or poor bone quality, a POP splint was applied to provide additional external support. Rehabilitation was initiated without delay; by the second postoperative day, patients began passive movements - primarily flexion and abduction of the shoulder and elbow - as tolerated by pain. Between 10 and 15 days postoperatively, patients progressed to more active exercises aimed at improving joint mobility and function. Rotational movements were introduced once radiographs showed evidence of soft callus formation, indicating early bone healing. Follow-up visits were conducted regularly in the outpatient clinic every four to six weeks, during which clinical evaluations, radiographic assessments, and rehabilitation guidance were provided. Once the fracture had healed radiographically, follow-ups continued every two to three months to track functional recovery and manage any remaining deficits. Patients were permitted to resume heavy lifting or strenuous activity only after a minimum of three months had passed and only when complete fracture union was confirmed on imaging. Those who exhibited restricted movement, stiffness, or delayed progress were referred to formal physiotherapy for customized rehabilitation programs that included mobility, strengthening, and functional training.

Statistical analysis

The collected data were entered into a Microsoft Excel spreadsheet, and statistical analysis was conducted using the IBM SPSS Statistics for Windows, Version 20 (Released 2011; IBM Corp., Armonk, New York, United States). The results were presented as mean ± SD, counts and percentages, and in table format.

## Results

From March 2023 to March 2025, 40 cases of shaft humerus fractures managed with intramedullary nails were followed up at Shri B M Patil Medical College, Hospital and Research Centre, BLDE (Deemed to Be University). The study’s observations were as follows: the mean age of patients with humeral shaft fractures was 46.5 years, with the youngest being 20 and the oldest 70 years old. The general characteristics of the study population are illustrated in Table 1.

**Table 1:** General characteristics The patients were categorized based on age, gender, the affected side, and the mechanism of injury. RTA: road traffic accident; H/O fall: history of fall

	No. of patients	Percentage
Age (years)		
20-30	8	20
30-40	7	17.50
40-50	7	17.50
>50	18	45
Sex		
Male	24	60
Female	16	40
Side		
Left	15	37.50
Right	25	62.50
Mode of Injury		
RTA	26	65
H/O fall	14	35

There were 26 (65%) type A fractures, six (15%) type B fractures, and eight (20%) type C fractures. A total of 36 (90%) patients experienced no intraoperative complications. The remaining patients had comminution in two (5%) cases and difficulties with reduction in two (5%) cases, as shown in Table 2.

**Table 2:** Intraoperative outcomes among the study population

Intraoperative complications	No. of patients	Percentage
Comminution	2	5
Difficult reduction	2	5
Nil	36	90

Most fractures, 36 (90%), united within 16 weeks. The duration of the union in weeks is presented in Table 3.

**Table 3:** Distribution of union times in weeks among the study population

Union in weeks	No. of patients	Percentage
10-12	12	30
13-16	24	60
16-20	2	5
Nonunion	2	5

Thirty-four (85%) patients had an uncomplicated outcome. One (2.5%) patient developed postoperative shoulder impingement, while another (2.5%) experienced shoulder stiffness and received physiotherapy. Two (5%) patients had non-union, for whom the nails were removed, and open reduction and internal fixation with plating and bone grafting were performed. One (2.5%) patient developed a superficial infection, which was treated with wound debridement, antibiotics, and regular dressings. Additionally, one (2.5%) patient had radial nerve palsy, which was evaluated and documented for muscle strength, as detailed in Table 4.

**Table 4:** Postoperative complications among the study population

Complications	No. of patients	Percentage
Shoulder impingement	1	2.50
Shoulder stiffness	1	2.50
Nonunion	2	5.00
Radial nerve palsy	1	2.50
Infection	1	2.50

The Disabilities of the Arm, Shoulder, and Hand (DASH) score was categorized as follows: less than 5 for excellent, 6 to 15 for good, 15 to 35 for fair/satisfactory, and more than 35 for poor. At the six-month follow-up, six (15%) of the 40 patients had an excellent outcome, 26 (65%) had a good result, and six (15%) had a fair outcome, as shown in Table 5.

**Table 5:** Association of age and mean DASH score DASH: Disabilities of the Arm, Shoulder, and Hand The mean DASH score was calculated for six months of follow-up.

	DASH	Score
Age (years)	Mean	SD
20-30	7.34	3.9
30-40	16.6	13.33
40-50	14.32	8.11
>50	21.43	10.78
Total	16.51	10.96

**Figure 8:**
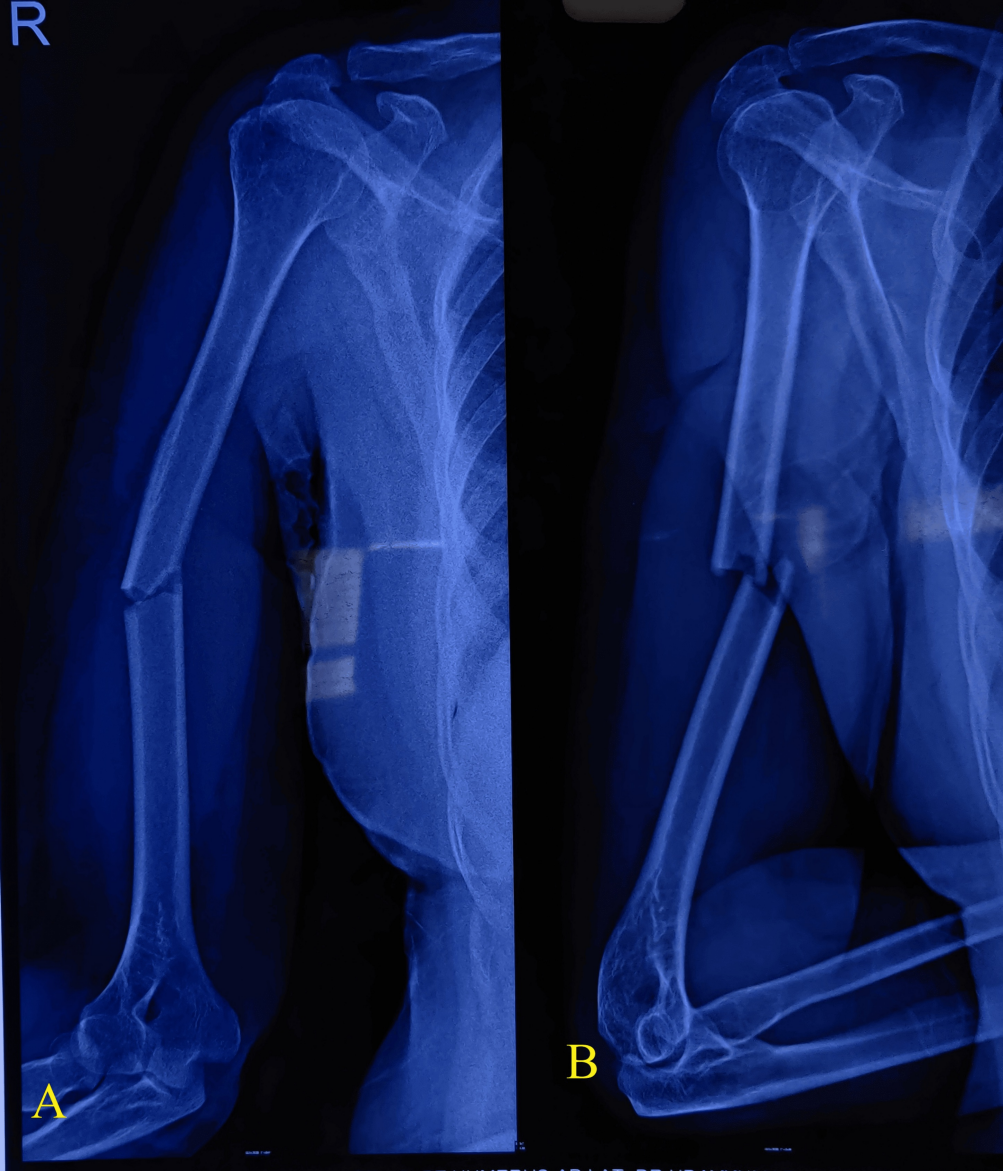
Radiographs showing a humerus shaft fracture Preoperative radiographs of a humeral midshaft fracture. (A) Anteroposterior view. (B) Lateral view showing the fracture line and alignment.

**Figure 9:**
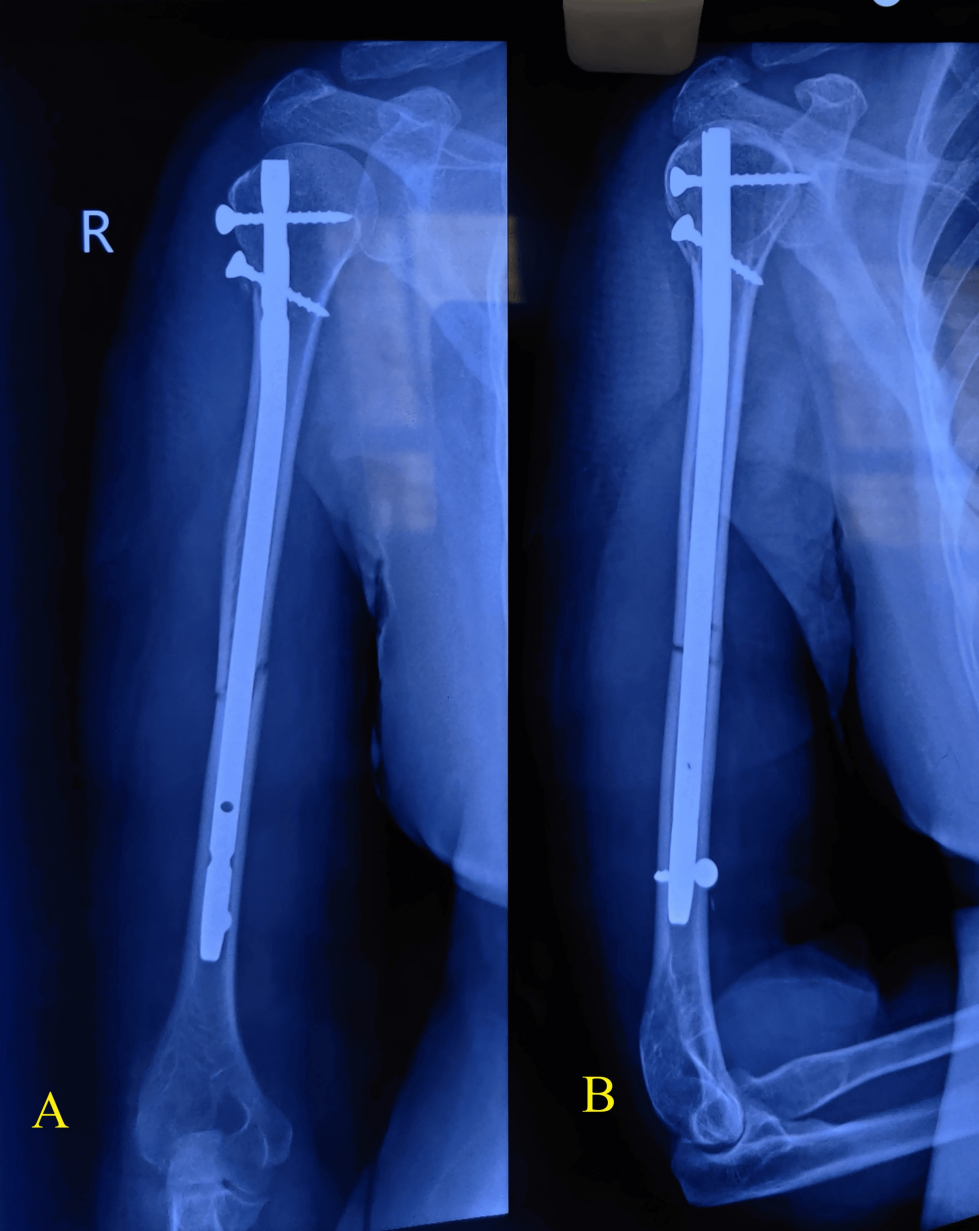
Radiographs showing a humerus shaft fracture treated with nailing Postoperative radiographs showing humeral shaft fracture treated with intramedullary nailing. (A) Anteroposterior view. (B) Lateral view demonstrating nail placement and fracture alignment.

The average DASH score was 16.5, with a standard deviation of 10.96. The highest mean score of 21.43 was observed in the age group over 50. The preoperative radiograph (Figure 8) illustrated the initial condition of the fracture, while the postoperative radiograph (Figure 9) showed satisfactory reduction and fixation following surgical intervention.

**Figure 10:**
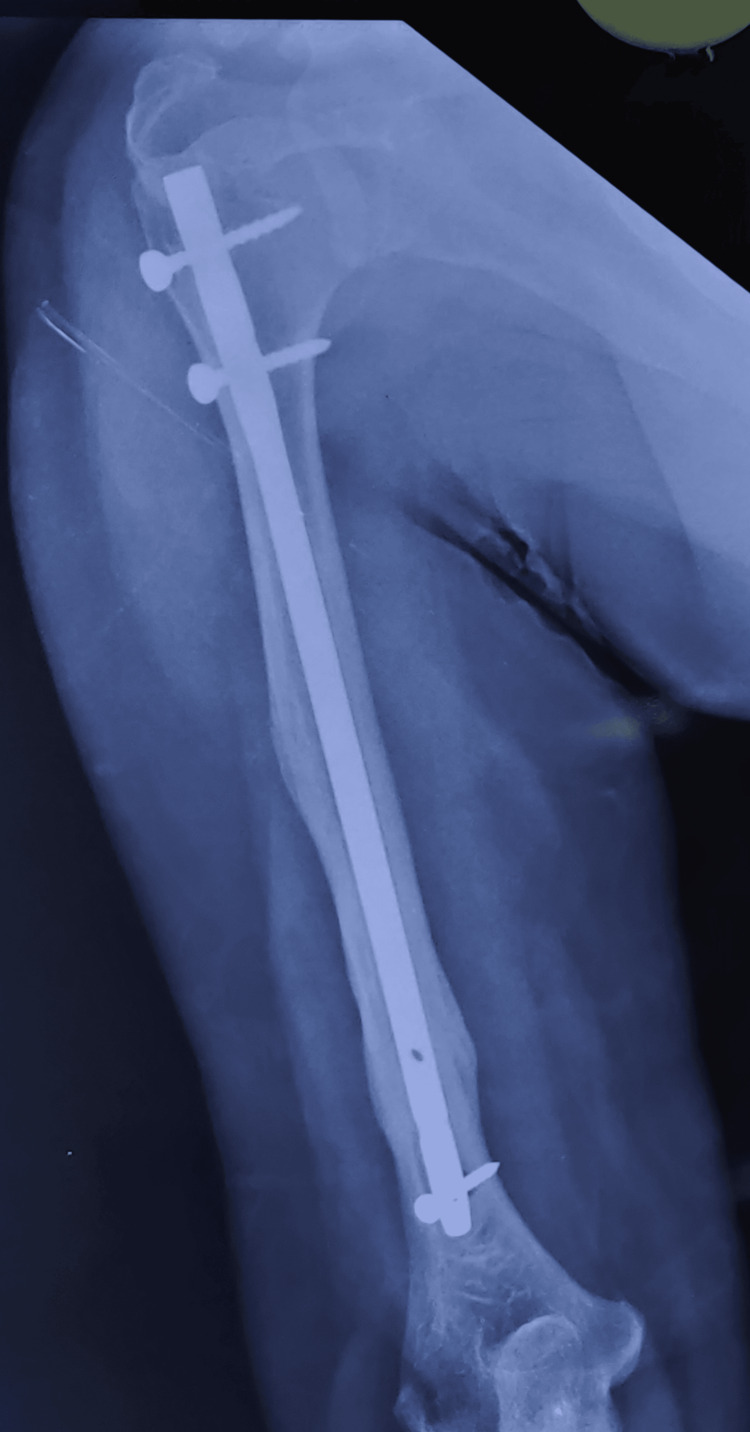
Radiographs of a midshaft humerus fracture treated with an intramedullary interlocking nail showing the anteroposterior view after a six-month follow-up

**Figure 11:**
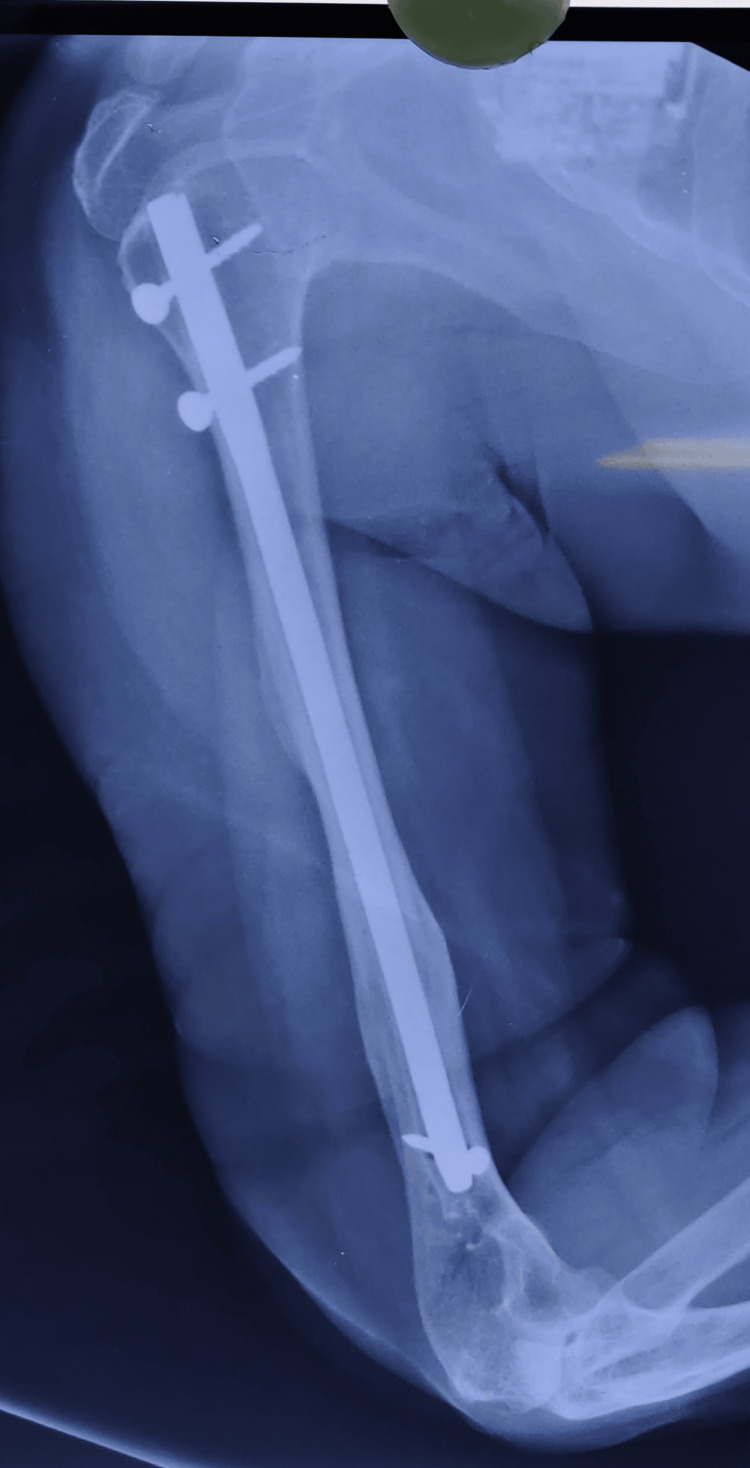
Radiographs of a midshaft humerus fracture showing the lateral view after a six-month follow-up

The postoperative radiographs taken at the six-month follow-up demonstrated a healed fracture site, as shown in Figures 10-11.

**Figure 12:**
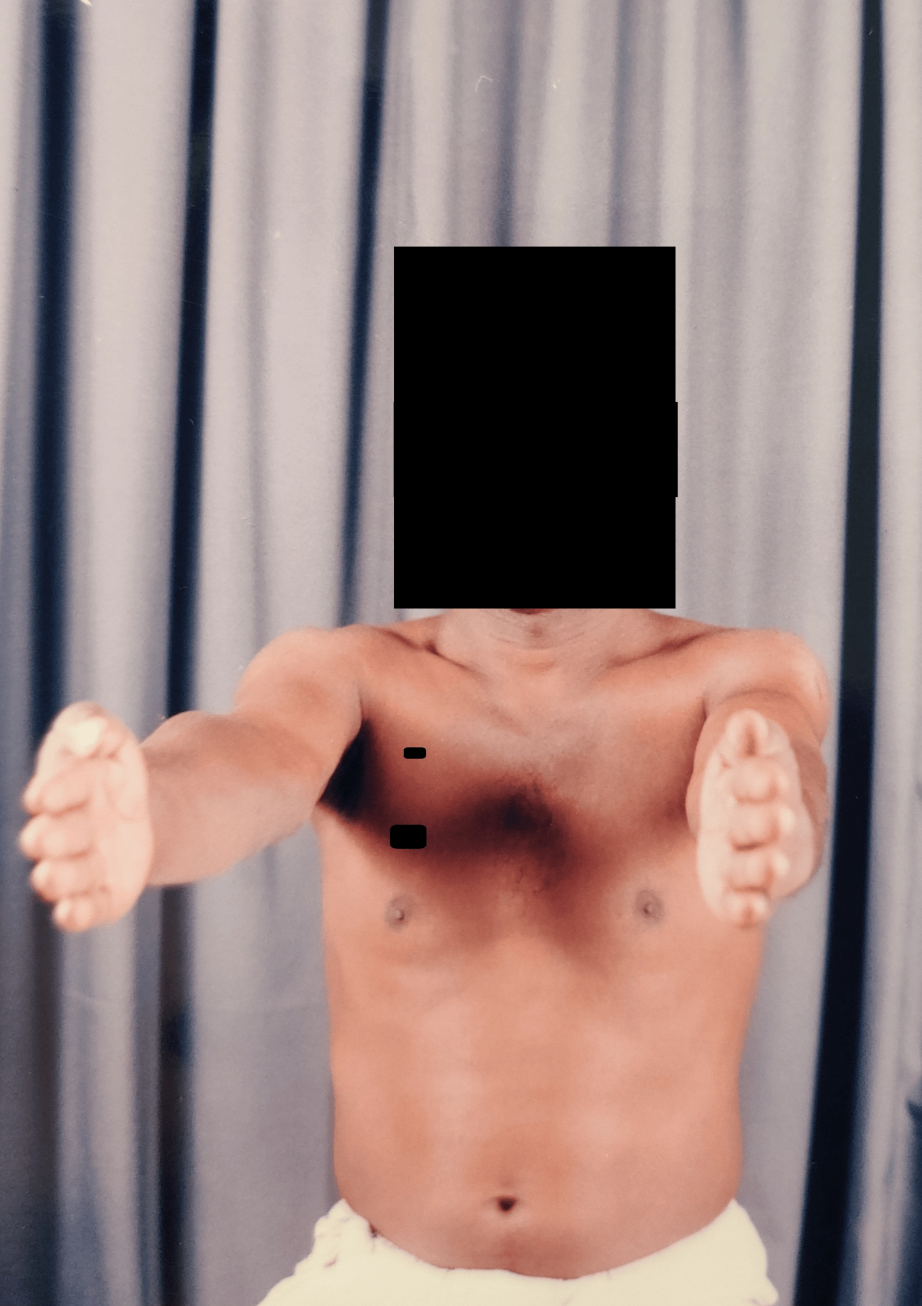
Shoulder forward flexion movements

**Figure 13:**
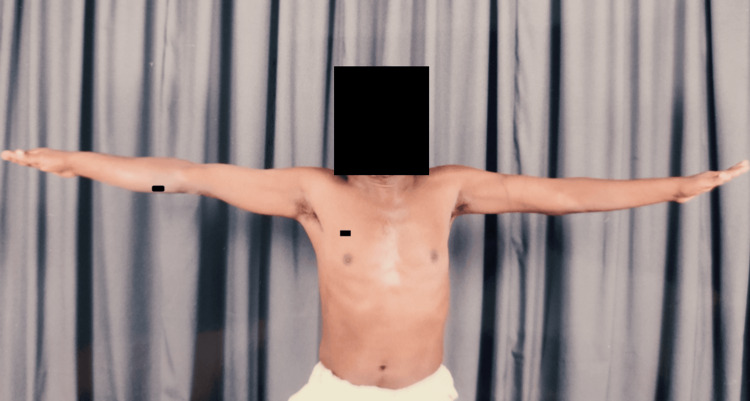
Shoulder abduction movements

**Figure 14:**
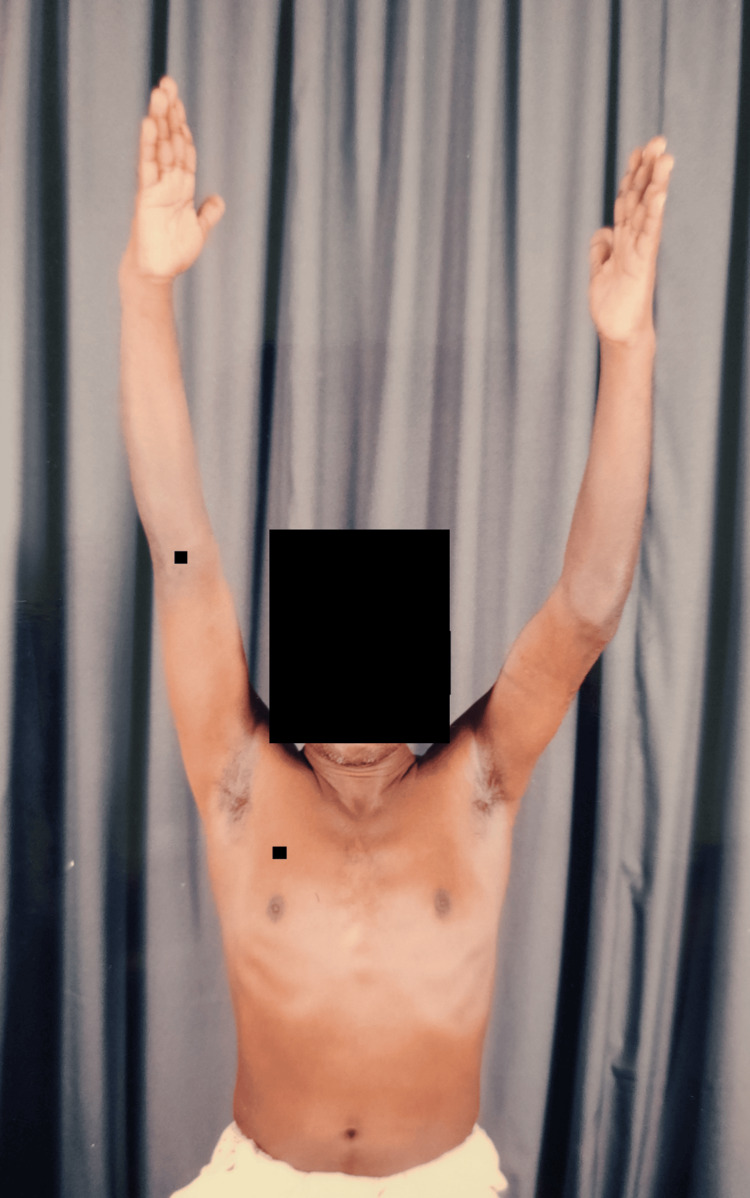
Shoulder overhead flexion movements

**Figure 15:**
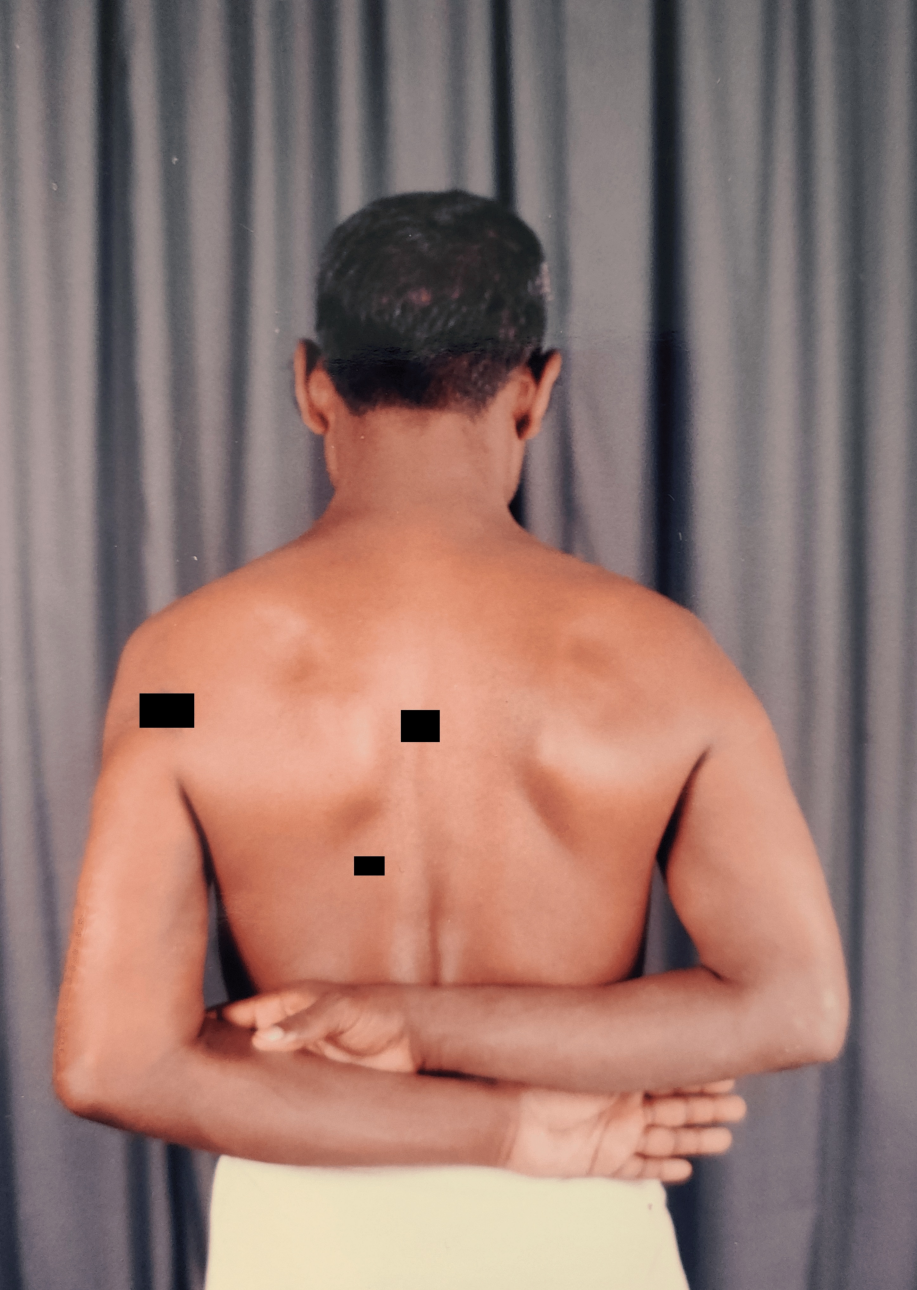
Shoulder rotation movements

Clinical images (Figures 12-15) illustrate full shoulder range of motion following surgery, highlighting the patient’s functional recovery.

## Discussion

Although conventional open reduction and internal fixation with plates and screws were considered the gold standard, there remained ongoing debates about the most appropriate treatment for humeral shaft fractures. The treatment plan accounted for the fracture pattern, bone strength, and the patient’s age. While plate fixation offered high union rates, it involved extensive surgery that required stripping soft tissues from the bone, which increased the risk of infection and nerve damage.

Plate fixation was less stable in osteoporotic bone, and healing tended to be slower. In contrast, IMIL nails preserved periosteal blood flow and the fracture hematoma, which are essential for fracture healing. Nailing provided biomechanical advantages over plating, offering load-sharing mechanical properties and relative stability, allowing micromotion at the fracture site. Using an IMIL nail also reduced the stress shielding effect and lowered the risk of refracture after implant removal [5]. Additionally, as an intramedullary implant, it was more suitable for osteoporotic bone.

This study included 40 patients with humeral shaft fractures at Shri B M Patil Medical College, Hospital and Research Centre, BLDE (Deemed to Be University).

Sex distribution

In Chandan et al.’s study [6], 15 (75%) patients were male. In our study, the majority of patients were male, 24 (60%), similar to McCormack et al.’s findings [7].

Mean age distribution

In this study, the average age of patients was 46.5 years, with the youngest being 20 and the oldest 70 years old. Additionally, 45% of patients were in their fourth or fifth decade. Similarly, the mean ages reported by McCormack et al. [7] and Putti et al. [8] were 40 and 38.75 years, respectively.

Mode of injury

In our study, 26 (65%) patients sustained their injuries from road traffic accidents (RTAs), while 14 (35%) had a history of falls (H/O Fall). This was similar to Chandan et al.’s study [6], where 25 (62.5%) patients experienced trauma from RTAs. In McCormack et al.'s study [7], 28 (63.63%) patients had injuries from RTAs, and Putti et al. [8] also identified RTA as a significant cause of fractures, accounting for 28 (82.35%) cases.

Side of the injury

Among the 40 patients with humeral shaft fractures who underwent surgery, 25 (62.5%) had fractures on the right side, while 15 (37.5%) had fractures on the left side.

Union rate

In most studies reviewed, most fractures healed within 16 weeks. This study found 36 (90%) fractures united within 16 weeks. This was comparable to the average union time of 13.4 weeks reported in the study by Suranigi et al. [9].

Union percentage

In this study, 38 patients (95%) achieved union, while two (5%) experienced nonunion. The union rates in Suranigi et al.’s study [9] were 51 (98%) patients.

Nonunion percentage

Previous studies reported that the incidence of nonunion following nailing ranged from 2% to 13%. In our study, two cases of nonunion were observed. Upon further inquiry, it was found that both patients were chronic smokers, and they underwent secondary procedures. The fractures eventually healed without additional complications.

Infection

In this study, one superficial infection (2.5%) was effectively treated with wound debridement and a three-week course of antibiotics.

Shoulder stiffness

This was the most common complication, as we observed one patient (2.5%) in our study. However, none of the patients experienced functional limitations as a result. Robinson et al. [10] found that five (17%) of their patients experienced this complication.

Radial nerve palsy

In our study, only one patient (2.5%) continued to experience postoperative palsy, which was monitored during each follow-up visit. The patient made a full recovery by the six-month follow-up. This rate was similar to the findings in Derbas et al. [11], where one (4%) patient experienced radial nerve palsy.

Shoulder impingement

In our study, one patient (2.5%) experienced shoulder impingement caused by nail prominence. After the nail was removed, the patient’s shoulder function fully recovered. This complication was noted in six (11.5%) cases in Suranigi et al. [9] and 12 (40%) in Robinson et al. [10]. We compared our study with the studies that were presented in Table 6.

**Table 6:** Comparison of findings Our study was compared with previous studies in terms of union rate, complications, and complication rate.

	Union rate (%)	Nonunion (%)	Infection (%)	Shoulder impingement (%)	Complication rate (%)
Our study	95	5	2.50	2.50	15
Suranigi et al. 2020 [9]	98	1.90	1.90	11.50	19
McCormack et al. 2000 [7]	89.48	9.50	4.70	14.20	-
Chandan et al. 2020 [6]	90	10	-	15.00	30

Functional outcome

In our study, IMIL nailing led to good to excellent functional outcomes in 80% of patients, with fewer complications than other internal fixation methods. The functional outcomes were measured using the DASH score, which had an average of 16.5 and a standard deviation of 10.96. In studies by Chandan et al. [6] and Zhang et al. [12], the mean DASH scores were 33.74 and 23.76 ± 16.78, respectively. Gerich et al.’s study [13] reported a mean DASH score of 25. We also examined the relationship between age, side of the fracture, intraoperative complications, union time, complications, and outcomes, but none showed statistical significance.

Study limitation

The primary limitation of this study was the relatively small sample size, which might have limited the generalizability of the findings. Additionally, with a small sample, the study did not fully capture the variability in patient outcomes or the incidence of less common complications. Furthermore, potential confounding variables such as patient social habits, adherence to rehabilitation protocols, and fracture patterns may have influenced the results, and these factors were not fully controlled for in the study design.

## Conclusions

Antegrade IMIL nailing was a safe and effective treatment for humeral shaft fractures, offering predictable union rates, shorter operative times, and low complication rates. Despite a few cases of shoulder stiffness and impingement, overall functional outcomes were good to excellent in most patients. Nevertheless, IMIL nailing remained a viable treatment option for humeral shaft fractures with an acceptable complication profile.
